# Multiomic Analyses of Dopaminergic Neurons Isolated from Human Substantia Nigra in Parkinson’s Disease: A Descriptive and Exploratory Study

**DOI:** 10.1007/s10571-021-01146-8

**Published:** 2021-09-15

**Authors:** Affif Zaccaria, Paola Antinori, Virginie Licker, Enikö Kövari, Johannes A. Lobrinus, Pierre R. Burkhard

**Affiliations:** 1 Neuroproteomics Group, University Medical Center, Faculty of Medicine, Geneva University, Geneva, Switzerland; 2 Department of Psychiatry, Geneva University Hospitals, Geneva, Switzerland; 3 Department of Pathology, Geneva University Hospitals, Geneva, Switzerland; 4 Department of Neurology, Geneva University Hospitals, Geneva, Switzerland

**Keywords:** Parkinson’s disease, Human brain tissue, Dopaminergic neurons, Laser capture microdissection, Transcriptomics, Proteomics

## Abstract

Dopaminergic neurons (DA) of the substantia nigra *pars compacta* (SN*pc*) selectively and progressively degenerate in Parkinson’s disease (PD). Until now, molecular analyses of DA in PD have been limited to genomic or transcriptomic approaches, whereas, to the best of our knowledge, no proteomic or combined multiomic study examining the protein profile of these neurons is currently available. In this exploratory study, we used laser capture microdissection to extract regions from DA in 10 human SN*pc* obtained at autopsy in PD patients and control subjects. Extracted RNA and proteins were identified by RNA sequencing and nanoliquid chromatography–mass spectrometry, respectively, and the differential expression between PD and control group was assessed. Qualitative analyses confirmed that the microdissection protocol preserves the integrity of our samples and offers access to specific molecular pathways. This multiomic analysis highlighted differential expression of 52 genes and 33 proteins, including molecules of interest already known to be dysregulated in PD, such as *LRP2*, *PNMT*, *CXCR4*, *MAOA* and *CBLN1* genes, or the Aldehyde dehydrogenase 1 protein. On the other hand, despite the same samples were used for both analyses, correlation between RNA and protein expression was low, as exemplified by the *CST3* gene encoding for the cystatin C protein. This is the first exploratory study analyzing both gene and protein expression of laser-dissected neuronal parts from SN*pc* in PD. Data are available via ProteomeXchange with identifier PXD024748 and via GEO with identifier GSE 169755.

**Supplementary Information:**

The online version contains supplementary material available at 10.1007/s10571-021-01146-8.

## Introduction

Parkinson’s disease (PD) is the most common neurodegenerative movement disorder, currently affecting about seven million people worldwide (Kalia and Lang 2015). Despite decades of extensive basic and translational research, PD remains an incurable condition, and the cause and mechanisms of the degeneration of dopaminergic neurons (DA) in the substantia nigra pars compacta (SN*pc*) remain to be fully elucidated. Since the emergence of high-throughput omics technologies some twenty years ago, several groups (Redensek et al. 2018), including ours, have been able to study the molecular profile of post-mortem SN*pc* samples with the purpose of identifying differential and specific molecular expression changes in PD compared to controls. Although these works allowed the in-depth molecular exploration of SN*pc* and the identification of altered signaling pathways such as inflammation (McGeer and McGeer 2004), oxidative stress (Jenner 2003), proteasome (McNaught et al. 2001), mitochondrial or cell iron pathways (Berg et al. 2001), there is still no consensus about the molecular cascade at the basis of nigral DA degeneration in PD brains. Failure to dissect these approaches more specifically could be mainly related to the nature of samples under study, i.e., the whole SN*pc* specimens that were compared between control and PD groups. Indeed, the molecular analysis of whole PD SN*pc* mainly involved glial cells owing to the PD-related dramatically reduced component of DA neurons, whereas the molecular analysis of control SN*pc* integrated a higher proportion of DA neurons, resulting in unbalanced and biased comparisons.

Thus, a first step toward a better understanding of nigral degeneration would require specific molecular analyses of DA neuronal regions from PD SN*pc*. In 2009, Simunovic et al. (2009) used laser capture microdissection (LCM) and RNA microarrays to analyze gene expression of dissected DA neurons from SN*pc* in PD samples. They identified a dysregulation of several known molecular regulatory pathways involved in PD pathogenesis such as oxidative stress-induced cell responses or dysfunction of the mitochondrial and ubiquitin–proteasome systems. However, this study, which focused on mRNA data only, revealed transcriptional activation of genes but did not inform about the protein expression level and function.

In 2016, the first proteomic study that focused on DA neurons was published by Plum et al. (2016). By combining LCM with nanoliquid chromatography mass spectrometry (nano-LC–MS/MS), they identify 1068 distinct proteins in DA neurons from healthy SN*pc* samples, but did not include PD samples in the study.

Therefore, to the best of our knowledge, there is still no published work applying quantitative proteomics to DA neuronal regions from SN*pc* samples in PD, or simultaneously applying both transcriptomic and proteomic workflows to the same samples in PD.

Over the last decade, the progressive improvements of LCM technology (Espina et al. 2007) in automation, velocity, and precision offer the opportunity to dissect frozen DA neuronal regions in conditions that are more suitable for relevant molecular analyses. The increased sensitivity of mass spectrometers and RNA sequencers enables comparative and quantitative multiomic approaches using low to very low amounts of biological material.

In this exploratory study, we used LCM to dissect DA neuronal regions from control and PD post-mortem SN*pc* specimens. In the first part, we used both qualitative transcriptomic and proteomic approaches, to confirm the integrity and validity of our samples, and the LCM-provided access to the specific protein content of DA neurons. This important quality control step led to the second part of this study, where a quantitative comparison of protein and gene expression by label-free approach and RNA sequencing (RNAseq), respectively, was performed in DA neuronal regions from control and PD samples. Importantly, the same specimens were used for both analyses. RNAseq analysis revealed 52 differentially expressed genes, and label-free proteomics highlighted 33 differentially expressed proteins in PD samples compared to matched controls. Transcriptomics and proteomics results were compared to identify the mRNA-protein couples for which the expression changes followed the same direction. This work is the first attempt to propose a multiomic analysis of DA neurons in the PD brain.

## Methods

### Human Brain Tissues

Ten frozen human midbrains, five from age-matched control patients, and five from PD patients were collected during 2 years by the Department of Clinical Pathology and Psychiatry of the Geneva University Hospitals under a procedure approved by the Geneva Ethical Committee (Table 1) and registered under the number CER 05-066 in accordance with the relevant guidelines and regulations. Written informed consent for brain autopsy and use for research was obtained from close family relatives. PD diagnosis was confirmed neuropathologically and controls, with no previous history of neurological or psychiatric disorders, were confirmed to be free of nigral abnormalities. Samples were cryopreserved at − 80 °C until further analysis.

**Table 1:** Summary for brain samples

Case ID	Primary diagnosis	Gender	Age (years)	PMI (h)	Proteomics	Transcriptomics
C1	Control	M	77	34	**x**	
C2	Control	M	85	31	**x**	
C3	Control	F	87	34	**x**	**x**
C4	Control	M	70	35	**x**	**x**
C5	Control	M	64	19	**x**	**x**
PD1	Parkinson's disease	M	79	17	**x**	
PD2	Parkinson's disease	M	84	38	**x**	**x**
PD3	Parkinson's disease	F	79	33	**x**	**x**
PD4	Parkinson's disease	M	73	25	**x**	
PD5	Parkinson's disease	M	73	25	**x**	**x**

*PMI* post-mortem interval

### Proteomic Analysis

#### Laser Capture Microdissection

12 µm tissue slices from each SN*pc* were cut at − 18 °C (Leica CM3050, Biosystems Switzerland AG, Muttenz, CH), mounted on 2 µm PEN membrane slides (Leica Biosystems Switzerland AG, Muttenz, Switzerland), fixed and dehydrated in ethanol. Collection of control and patient DA neurons was alternated to avoid a time-related bias.

DA neuronal regions were visually identified by their brown neuromelanin pigment under bright field microscopy on a Leica LCM6000 instrument (Leica Microsystems GmbH, Wetzlar, Germany). Approximately 2050 regions of DA neurons were accurately delimited at × 200 magnification to reduce contamination by surrounding tissue, microdissected, and catapulted into the vial cap in 8 µl of RapiGest™ 0.1% (Waters, GmbH, Milford, MA, USA) in TEAB 0.1 M (Sigma-Aldrich Inc., St. Louis, MO, USA). The vial was vortexed upside-down, centrifuged to recover the sample at the bottom, and sonicated with a VialTweeter UIS250v (Hielscher Ultrasonics GmbH, Teltow, Germany) to foster lysis and DA-neuron detachment from the PEN membrane (70% amplitude, 0.5 s cycle, 20 bursts, 5 times, on ice between each cycle). Samples were stored at − 80 °C.

#### Proteomic Analysis with Mass Spectrometry

Microdissected DA neuronal regions were thawed simultaneously, the volume was adjusted to 100 µl with lysis buffer (RapiGest™ 0.1% Waters, Corporation, Milford, MA; TEAB 0.1 M; Sigma-Aldrich, St. Louis, MO), and protein concentration was estimated with a NanoDrop™ 2000 spectrophotometer (Waltham, Massachusetts, USA). For trypsin digestion, the proteins were treated with TCEP 1 mM (Sigma-Aldrich, St. Louis, MO) (1 h at 60 °C; Sigma-Aldrich, Saint-Louis, US-MO) and iodoacetamide 4 mM (30 min at room temperature in the dark, shaking at 250 rpm, Sigma-Aldrich, St. Louis, MO), and trypsin (porcine, Promega Corporation, Madison, WI) was added to samples in a 1:25 ratio overnight. The reaction was stopped with 10% FA. RapiGest™ was removed by acid precipitation after incubation at 37 °C for 40 min and centrifugation at 13,000 rpm for 20 min. The supernatant with the peptides was cleaned with a C_18_ microspin column (Harvard Apparatus, Holliston, MA) according to the manufacturer instructions, dried under speed-vacuum, and stored at − 80 °C.

MS analysis was performed according to the protocol of the Proteomics Core Facility of the University of Geneva (https://www.unige.ch/medecine/proteomique/), as previously described (Dor et al. 2019).

Peptide digests were solubilized in 5% acetonitrile and analyzed by electrospray ionization on a linear trap quadrupole (LTQ) Orbitrap velos Pro (Thermo Scientific, San Jose, CA, USA) equipped with a NanoAcquity system (Waters, Milford, MA, USA). Peptides were trapped on a home-made 5 μm 200 Å Magic C_18_ AQ (Michrom) 0.1 × 20 mm pre-column and separated on a commercial 0.075 × 150 mm Nikkyo (Nikkyo Technology, Tokyo, JPN) analytical nanocolumn (C_18_, 5 μm, 100 Å). The analytical separation was run for 54 min (flow rate 200 nl/min) using a gradient as follows: 0–1 min 95% A (0.1% FA) and 5% (99.9% acetonitrile, 0.1% formic acid) then to 65% A and 35% B for 55 min, and 20% A and 80% B at 65 min. For MS survey scans, the orbitrap (OT) resolution was set to 60,000 and the ion population was set to 5 × 105 with an *m*/*z* window from 400 to 2000.

Three gas-phase fractions (GPF) for data-dependent MS/MS selection were defined in the following *m*/*z* ranges: 400–598, 593–746, and 741–2000 Th (Scherl et al. 2008).

Five precursor ions were selected for collision-induced dissociation (CID) in the LTQ. The ion population was set to 1 × 104 (isolation width of 2 m/*z*), while for MS/MS detection in the OT, it was set to 1 × 105 with an isolation width of 2 m/*z* units. The normalized collision energies were set to 35% for CID.

#### Data Analysis for Proteomics

MaxQuant (version 1.5.8.3) was used to process Thermo raw files. For protein identification, data were searched against the UniProtKB/Swiss-Prot human database (release 2018_05, with 26,336 protein entries). N-terminal protein acetylation and methionine oxidation were set as variable modifications and cysteine carbamido methylation as fixed. The default parameters were used for the instrument choice. Only one missed cleavage was allowed and search for second peptide matches and match between runs were activated. Peptides and protein FDR was set to 0.01. For protein quantification, label-free quantification (LFQ) was chosen with a min. ratio count of 1 and unique + razor peptides were used. The other parameters were left as defaults.

Data analysis was performed using Perseus software. Common contaminants were filtered out and LFQ protein intensities were log_2_ transformed. At least 70% of protein intensities were required overall before imputing the missing values from a normal distribution. LFQ intensities were averaged across technical replicates before performing a two-sample *t*-test. Proteins with a *p*-value < 0.05 and a fold change > 1.5 were considered differentially expressed between patients and controls.

The MS proteomics data have been deposited to the ProteomeXchange Consortium via the PRIDE partner repository (Perez-Riverol et al. 2019) with a dataset identifier PXD024748. The description of each submitted file is detailed in Supplementary Information SI-4.

### Gene Expression Analysis

#### LCM for Gene Expression Analysis

For gene expression analysis, we used three PD samples and three controls, for which SN*pc* was still available after proteomic sample preparation.

12 µm tissue slices from each substantia nigra were cut at − 18 °C and processed as described in the proteomic section. Approximately 70 regions of DA neurons were dissected in duplicates for each of the six different samples and collected by gravity in distinct vials. The 12 resulting groups of DA neuronal regions were quickly frozen on dry ice and stored at − 80 °C.

#### RNA Extraction for Quality Control

Tissue depleted of DA neuronal regions after LCM was also collected from the slides in 100 µl of lysis/denaturing buffer from the RNAqueous micro kit (Life Technologies, Zug, Switzerland). RNAs were extracted following the manufacturer protocol, quantified with a Qbit™ fluorometer (Thermo Fisher, Waltham, MA, US), and analyzed with an Agilent 2100 Bioanalyser (Agilent Technologies, Palo Alto, CA) to check the RNA profile and obtain the RNA integrity number (RIN).

#### RNAseq Library Preparation and Sequencing of NM-Granules

The SMARTer™ Ultra Low RNA kit from Clontech was used for the reverse transcription and cDNA amplification according to the protocol described by Vono et al. (2019), starting with 70 cells as input. Samples were defrozen simultaneously and solubilized in 10 µl of lysis buffer. After reverse transcription and amplification, 200 pg of cDNA were used for library preparation using the Nextera XT kit from Illumina. Library quality and molarity were assessed with the Qbit and Tapestation using a DNA High sensitivity chip (Agilent Technologies). Pools of 12 libraries were diluted at 2 nM for clustering on a Single-read Illumina Flow cell. Reads of 50 bases were generated using the TruSeq SBS chemistry on an Illumina HiSeq 4000 sequencer at the iGE3 Genomics Platform of the University of Geneva (https://ige3.genomics.unige.ch).

#### RNAseq Data Analysis

Sequencing quality control was performed with FastQC (v.0.11.5). Sequencing data were mapped to the UCSC human hg38 reference genome using STAR aligner (v.2.5.3a). The transcriptome metrics were evaluated with the Picard tools (v.1.141) and informed the decision to exclude two samples due to a low number of reads assigned to a gene.

The differential expression analysis PD/controls was carried out with the statistical Bioconductor package edgeR (v.3.14.0). The gene counts were normalized according to the library size. The genes having a count above 1 count per million reads (cpm) in at least two samples were carried forward for the analysis. The list of 26,485 genes was reduced to 22,561 after filtering out the poorly or not expressed genes. The differentially expressed gene tests were done with a GLM (general linear mode) with a negative binomial distribution. *p*-values were corrected for multiple testing error with a 5% FDR using the Benjamini–Hochberg procedure to retain only the significant genes.

In order to check whether the protein product of the differentially expressed genes has been already detected by MS, we generated a list of brain proteins with Nextprot (Gaudet et al. 2017) using the Advanced search (SPARQL) tool and querying for human proteins identified in the brain by MS with two distinct peptides seven or more aminoacids long.

Gene expression data have been deposited on Gene Expression Omnibus (GEO) under the identifier GSE 169755.

## Results

### Integrity and Quality of Samples by Transcriptomics

Before proceeding to the quantitative comparisons between PD and control samples, we controlled that our sample preparation protocol preserved extracted molecules in sufficient quality for omics analyses. As RNAs are known to be more vulnerable entities than proteins, we used transcriptomic approaches to analyze RNA quality of our samples, by different ways, at different steps of the workflow.

**Fig. 1:**
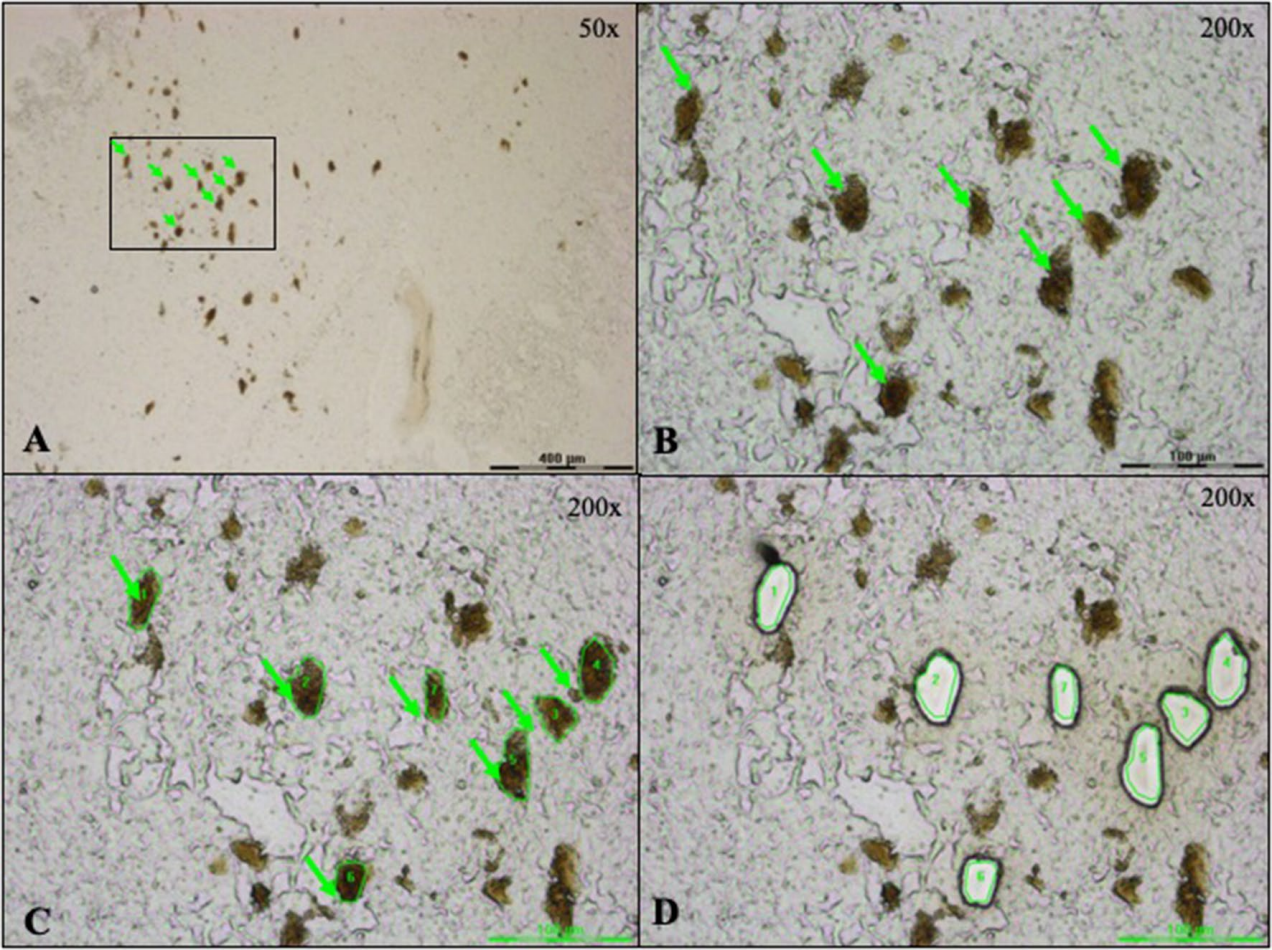
LCM capture of DA neurons from a section of substantia nigra tissue mounted on a PEN membrane slide. **A** DA neurons (pointed by the green arrows) can be visually identified by their brown pigment (× 50 magnification). The black rectangle highlights the region depicted in figures (**B**–**D**) at × 200 magnification. **C** The green lines define the DA neurons to guide the laser beam. **D** The shapes appear empty after cutting and collecting the granules in the tube cap situated under the slide

**Fig. 2:**
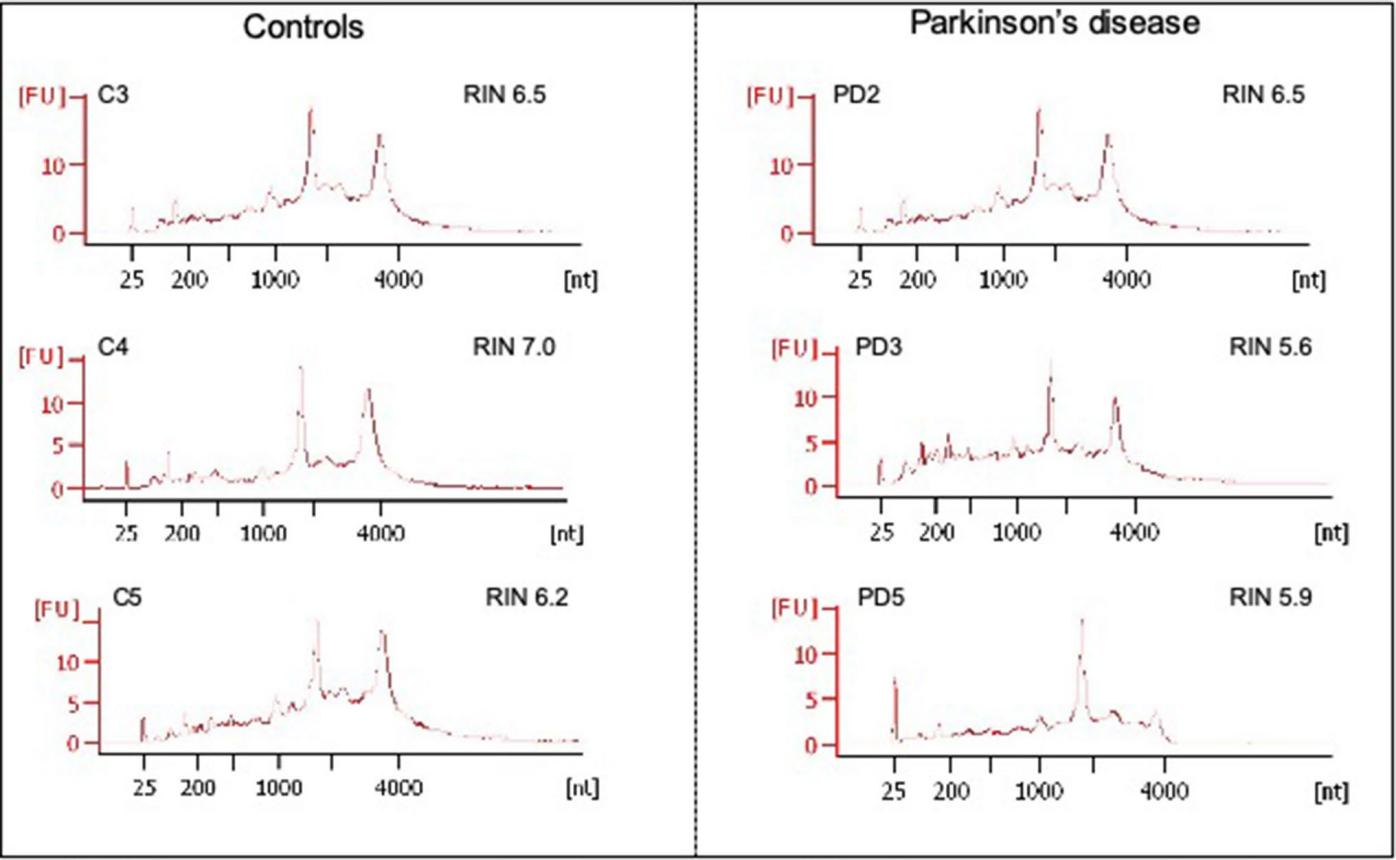
Quality control of RNA extracted from DA neurons in PD and control samples. The electrophoretic profiles and the resulting RNA integrity number (RIN) were obtained to confirm integrity of all samples and were compared between control and PD samples

To this purpose, tissue slices from SN*pc* of controls and PD patients (Table 1) were mounted on slides for LCM of DA neuronal regions (Fig. 1). About 70 zones of DA neurons, per sample, were microdissected in duplicates and collected in distinct vials. For each sample, after dissection, we collected on slide the remaining tissue into lysis buffer, extracted RNAs, and determined their quality through observation of their electrophoretic profiles and the RIN measurement (Fig. 2). The electrophoretic profiles revealed an average RIN of 6.0 and 6.6 for PD and control samples, respectively. And although it showed decreased 18S and 28S peak intensity, peaks were clearly visible and positioned at the right nucleotide size (Fig. 2; Supplemental Table 1). In this context, we considered RNA quality as good enough to proceed to cDNA amplification with the SMARTer™ Ultra Low RNA kit. Starting with an average of 70 regions of DA neurons per sample, the cDNA concentration obtained after amplification was homogeneous across all samples with an average cDNA concentration of 0.15 ± 0.01 ng/µl in PD samples and 0.16 ± 0.04 ng/µl in control samples and a global average cDNA concentration of 0.15 ± 0.03 ng/µl (Supplemental Table 2). 200 pg of cDNA were used to generate one library for each individual sample. The average fragment size was 300 bp and the fragments distribution was homogeneous across all samples, with no significant difference between control and PD groups (Supplemental Table 2). Altogether, these results validated the sufficient quality and homogeneity of our samples, two important aspects before initiating quantitative comparisons between control and PD groups.

### Assessment of LCM Specificity by Proteomics

In order to validate the capacity of our protocol to specifically highlight the molecular content of DA neuronal regions, we performed a proteomic analysis of this region collected from five control samples and five PD samples (Table 1). To obtain a sufficient amount of protein extract to perform triplicate injections for three GPFs^11^ for data-dependent MS/MS selection, we dissected at × 200 magnification an average of 2050 regions of DA neurons per sample (Supplemental Information 1), covering an average area of 750,000 µm^2^. To obtain this quantity of biological material, an average of 16 and 37 tissue sections were LCM-processed for control and PD samples, respectively.

**Fig. 3:**
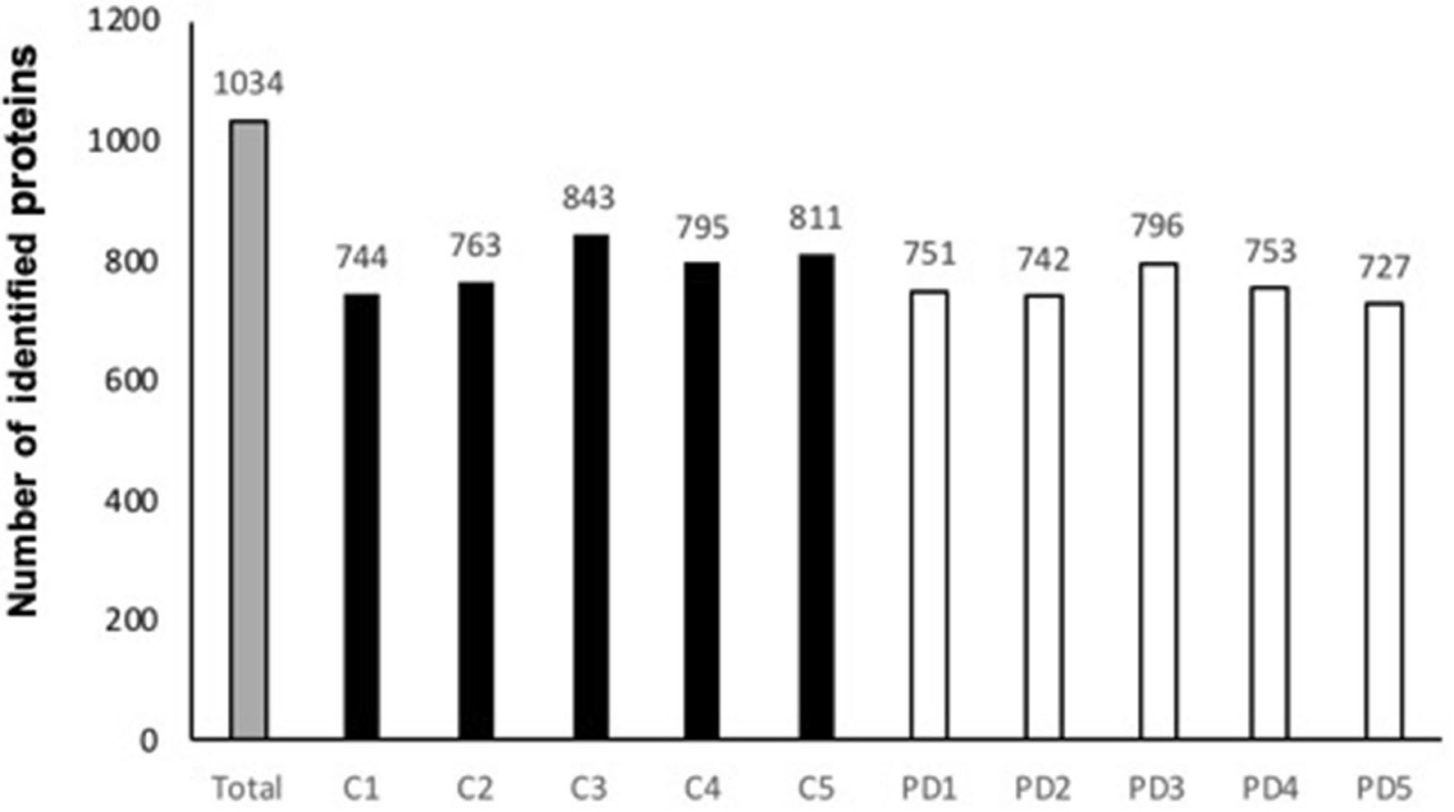
Number of proteins identified in DA neurons of post-mortem SN*pc* by nano-LC–MS/MS: across all analyzed samples (Total), in control samples (C1 to C5) and Parkinson’s disease samples (PD1 to PD5)

The total amount of proteins extracted from these neurons ranged from 18 to 24 µg. 6 µg proteins of each sample were trypsin digested and injected in triplicates for three GPF runs with nano-LC–MS/MS. Data analysis with MaxQuant allowed the identification of 727 to 843 distinct proteins (Fig. 3). The comparison of these 10 protein-lists highlighted a total of 1034 distinct proteins, identified by at least two proteotypic peptides (SI-1).

**Fig. 4:**
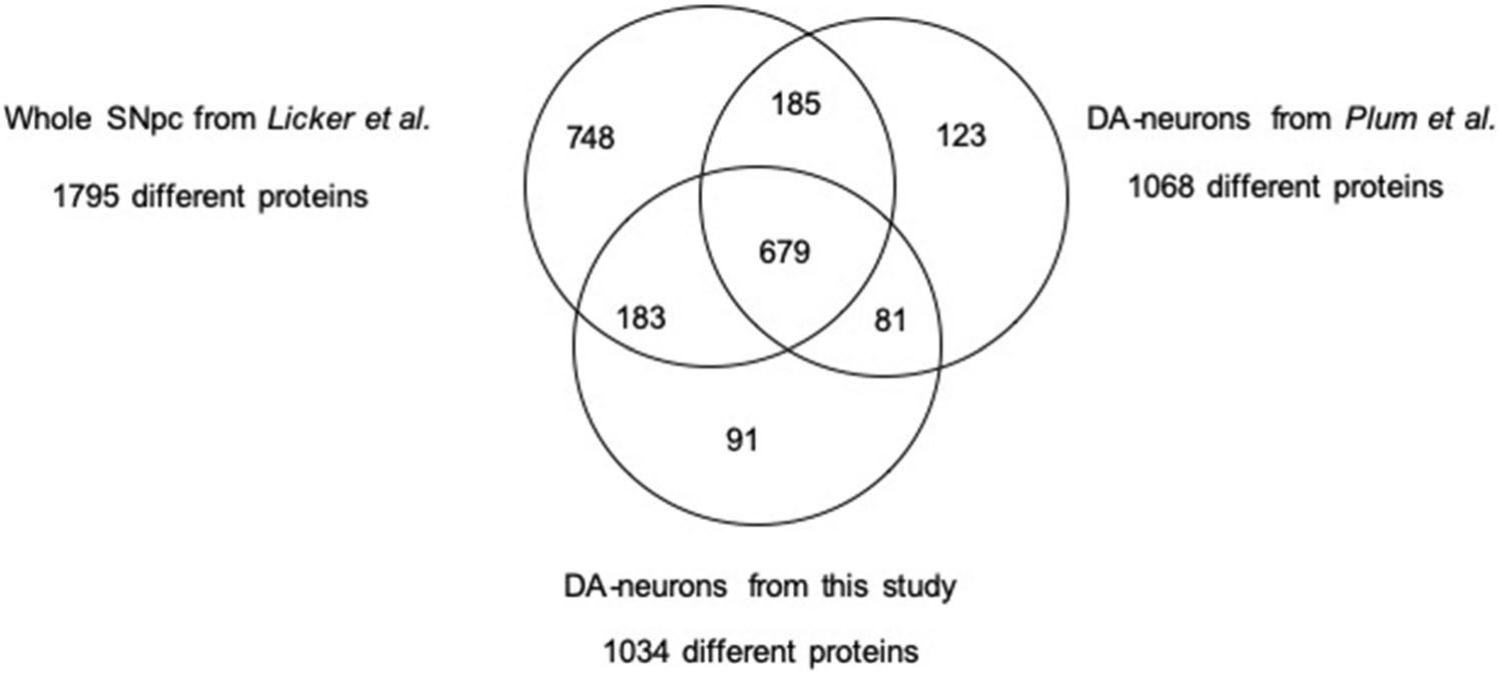
Qualitative comparison of proteins identified from DA neurons with whole SN*pc*. Venn diagram representing both common and specific proteins identified in whole SN*pc* and DA neurons

To confirm the quality of our DA-neuron enrichment using LCM approach, we compared our protein list with the list published by Plum et al. (2016)*.* These authors identified 1068 distinct proteins, a figure very similar to our study. Interestingly, there was a 74% overlap between the two lists. In fact, 760 of the 1034 proteins were identified in both studies. Then, to demonstrate that dissection of DA neuronal region, a subcompartment of SN*pc*, gave access to a specific subproteome, we compared our 1034 proteins with the most exhaustive proteome of whole SN*pc*, published by our group in 2014 (Licker et al. 2014), with a list of 1795 different species (Fig. 4 and SI-3). On the one hand, among the 1034 proteins identified into dissected DA neurons, 862 species were also identified into the whole SN*pc*. On the other hand, 170 proteins were only present into the DA-neuron compartment (SI-3). In fact, while these 170 proteins were identified in at least 80% of DA-neuron samples, they were never identified into the whole SN*pc* samples. Interestingly, the comparison of the whole SN*pc* with Plum et al. (2016) revealed 864 common proteins, a number very similar to our study. And among the 170 exclusive proteins, we only identified in the current list, and 80 were also identified by Plum et al. (2016). These qualitative observations and comparisons with previous published studies suggest that our LCM-nano-LC–MS/MS protocol allowed access to a specific proteome of DA neuronal regions, which, as anticipated, is not accessible with whole SN*pc* approaches.

In this first part of the study, we used transcriptomic and proteomic approaches (i) to confirm that our LCM-related sample preparation preserved samples in sufficient quality for molecular analyses and (ii) to validate that subcellular selection of DA neurons offered access to a specific subproteome. These results strengthened the interest for quantitative multiomic approaches to identify PD-related specific events in DA neurons.

### Differential Expression Between Control and PD Samples

To proceed to comparative analyses between PD and control samples through multiomic workflows, we first compared the mRNA abundance of 17,002 protein-coding genes between PD and control in DA neuronal regions (SI-2). A total of 52 genes (0.3%) showed significantly different gene expression at FDR *p* values < 0.05. In PD samples, RNA expression was increased for 40 genes and decreased for 12 genes (Table 2). Among these 52 differentially expressed genes, at least 10 genes are of particular interest: the upregulation of *MT1H*, *CXCR4*, *PNMT*, *BTG3*, *LRP2*, *AGT*, *S100B*, *MAOA* and *CST3* and the downregulation of *CBLN1* have been observed in previous studies investigating PD or other neurological disorders. Upregulated genes showed differences ranging from 3-fold change for *MAOA* to 98-fold change for *MT1H*, while *CBLN1* was downregulated with a 2.5-fold change.

**Table 2:** Differentially expressed genes between PD and control samples

Genes highlighted in gray were upregulated in our study. Genes highlighted in white were downregulated in our study. Genes marked with an asterisk (*) have been already reported as dysregulated in PD

Second, we compared protein expression between PD and control groups using label-free quantification (LFQ). Among the 1034 identified proteins, 33 (3.2%) were differentially expressed (*t*-test, *p* value < 0.05) between PD and control, with at least a 1.5-fold change (Table 3), including 12 proteins with increased and 21 with decreased expression in PD samples. Among these 33 differentially expressed proteins, three upregulated, cystatin-C, cathepsin L1, Annexin A2, and two downregulated, Aldehyde dehydrogenase 1 and alpha-1-antitrypsin, proteins in PD samples deserve a particular attention as they also appeared dysregulated in previous publications involving PD or other neurological disorders. PD-overexpressed proteins showed differences ranging from 1.8-fold change for cystatin-C to 3.5-fold change for vimentin, while downregulated proteins showed differences ranging from 2.5-fold change for aldehyde dehydrogenase 1 to 3.7-fold change for alpha-1-antitrypsin.

**Table 3:** Differentially expressed proteins between PD and control samples

Proteins highlighted in gray were upregulated in our study. Proteins highlighted in white were downregulated in our study. Proteins marked with an asterisk (*) have been already described in PD as dysregulated

Somewhat surprisingly, correlation of transcriptomic and proteomic analyses only revealed one common event: the *CST-3* gene and its corresponding translated protein cystatin-C that were both significantly upregulated in PD samples. That was not the case for *S100 B* and *MAOA*, 2 upregulated genes in our study, as expression of their corresponding protein was not significantly different in PD samples. Concerning the six others interesting genes (*MT1H*, *CXCR4*, *PNMT*, *BTG3*, *LRP2*, *AGT*), their corresponding proteins were not identified by our proteomic workflow. To better understand the low correlation between transcriptomic and proteomic data, we focused on the proteins identified and quantified in our proteomic workflow and present in the list of the 52 differentially expressed genes. In fact, only 7 gene-related proteins (13.5%) were identified by nano-LC–MS/MS among the potential 52 gene products, whereas no corresponding protein for the 45 remaining dysregulated genes could be found, making correlation between transcriptomic and proteomic data impossible. Among these 52 genes, 19 had never seen their corresponding protein identified by MS from brain samples according to Nextprot database (Table 2).

In summary, this second part of the study was devoted to compare for the first time RNA and protein expressions from DA neurons, in PD and control SN*pc*. These comparative analyses separately revealed relevant differences of expression in PD samples, supporting previous observations conducted in whole SN*pc* studies. However, correlation between transcriptomic and proteomic data was limited by our proteomic workflow. In fact, while the transcriptomic approach provided information about approximately 15,000 genes, the proteomic approach was limited to 1000 proteins. Moreover, the proteins corresponding to the majority of dysregulated genes were not identified by our nano-LC–MS/MS-related workflow.

## Discussion

The difficulty to identify key molecular mechanisms at the basis of PD is a major obstacle to the development of neuroprotective therapies. DA neurons in the SN*pc* represent the main cellular compartment affected by degeneration in PD and thus appear as relevant entities to isolate and analyze.

In this study, we used LCM to extract DA neuronal regions from post-mortem control and PD SN*pc*. RNA-based analysis confirmed sufficient quality of all used samples for molecular analyses. A qualitative proteomic analysis of our samples showed high similarity with Plum et al. (2016) who, using LCM-coupled nano-LC–MS/MS, provided an exhaustive proteome of DA neurons from healthy subjects. Our present study confirms the feasibility and the relevance of such workflow and updates the human proteome of DA neurons with new identified proteins. Moreover, the comparison of our list with the whole SN*pc* proteome published by our group (Licker et al. 2014) confirmed that using LCM allows access to a specific subproteome, here composed of 170 species, which were not identified in the whole SN*pc* samples despite a protein fractionation protocol. These 170 proteins also update the human proteome of the SN*pc*.

We then applied both quantitative proteomic and transcriptomic workflows to our dissected DA neuronal regions in order to identify specific molecular events in PD-related samples. To our knowledge, we are the first (1) to compare protein expression of DA neuronal regions in PD and control samples and (2) to apply both proteomic and transcriptomic workflows from these samples. The real challenge to perform this kind of comparative analysis relies on the high number of tissue sections required for PD samples. In fact, for each PD sample, an average of 37 tissue sections were microdissected in order to collect enough DA neuronal regions. In total, more than 300 tissue sections were required for this multiomic study. This information reflects the significant DA-neuron loss observed in PD samples and thus the highly challenging context to perform these experiments.

In our study, the comparative analysis of gene expression revealed 52 dysregulated entities in PD samples, among which *LRP2* was upregulated. *LRP2* encodes for megalin receptor, also known as the neuronal receptor for metallothionein proteins, proteins whose function as metal exchanger would be neuroprotective for brain tissue. In PD context, gene expression of *LRP2* has been previously reported to increase in nigral DA neurons (Michael et al. 2011).

*PNMT* encoding for phenylethanolamine *N*-methyltransferase was also upregulated in our study. Interestingly, phenylethanolamine *N*-methyltransferase can induce, through its catalytic activity, cytotoxic N-methylated beta carbolineum cations, which have structural and functional similarity with neurotoxic 1-methyl-4-phenyl-pyridinium cation (MPP+). Several studies have shown that within DA neurons, PNMT-induced beta carbolineum cations inhibit mitochondrial respiration (Drucker et al. 1990; Matsubara et al. 1998). High PNMT catalytic activity has been observed in SN*pc* and locus coeruleus, the two most affected brain areas in PD (Kopp et al. 1979). Thus, our results confirm previous observations and strengthen the hypothesis suggesting that increased levels of PNMT could induce neurotoxin-mediated death (Gearhart et al. 2002).

In PD brain, increased activation of microglia releases pro-inflammatory molecules such as cytokines and may contribute to neuronal damage observed in this disorder. Among cytokines, CXCR4 and its ligand CXCL12 are important members of the chemokine family and are expressed in the central nervous system. In 2009, Shimoji et al*.* demonstrated that CXCR4 was elevated in SN*pc* DA neurons, more in PD than in control samples (Shimoji et al. 2009). In the same study, the authors also suggested that increased CXCR4 expression occurs before and is not consecutive to DA neuronal loss. Thus, CXCR4 signaling would enhance the loss of DA neurons. In our study, we observed the upregulation of *CXCR4* gene expression in PD samples, confirming results from previous studies and the important role of inflammation in PD degeneration.

The enzyme monoamine oxidase A (MAOA) is a drug target in the treatment of PD (Miklya 2016). The inhibition of MAO by drugs prevents dopamine breakdown, maintaining a higher level of dopamine into the brain of PD patients. MAOA is principally located in neurons and is primarily responsible for dopamine metabolism in the latter (Levitt et al. 1982). In 2017, Tong et al*.* observed a 33% increase of the protein expression of MAOA in PD-related whole SN*pc* (Tong et al. 2017). Considering that MAOA is mainly expressed in dopamine neurons, which are reduced in PD conditions, they were surprised by these observations and proposed different explanations including the expression of MAOA by glial cells or an upregulation of MAOA into surviving DA neurons.

In our present study, we observed an increased expression of *MAOA* gene in PD DA neurons supporting an upregulation of MAOA into surviving DA neurons, although we cannot entirely exclude contamination by others cells. These results confirm previous observations and strengthen the interest toward MAO inhibitors for symptomatic purposes.

In our study, we observed a downregulation of *CBLN1*, that encodes for cerebellin 1 protein, in PD samples. In 2018, Zucca et al*.* confirmed the expression of cerebellin-1 protein into DA neurons (Zucca et al. 2018).

*CBLN1* is among the most consistently reported downregulated genes across studies on PD (Grunblatt et al. 2004; Moran et al. 2006). Cerebellins are hexameric protein hormones with neuromodulator functions. Their physiological role is not entirely elucidated although it has been reported that cerebellins increase norepinephrine synthesis. Consequently, when not enough cerebellin is present in the brain, the level of dopamine might also decrease.

All these dysregulated genes have been previously described in others studies and are particularly interesting according to the function of the corresponding proteins. Unfortunately, the quantitative expression of these corresponding proteins could not be measured in our samples. In fact, while RNAseq provides a complete picture of all expressed transcripts and because low copy mRNAs are also amplified during the workflow, protein identification using non-targeted MS-related proteomics is limited by instrument-related dynamic range. Indeed, for 19 out of the 52 dysregulated genes, the protein product has never been identified by MS approaches. Moreover, among the 1034 identified and quantified proteins, only 7 were encoded by genes we observed as dysregulated in our study. At first glance, the low correlation between transcriptomic and proteomic data may seem odd but several previous studies have already confirmed this trend (Greenbaum et al. 2003). For example, in 2016, Dumitriu et al. (2016) compared RNA and protein expression from post-mortem human prefrontal cortex in PD and control samples. Although 283 proteins and 1095 mRNAs were significantly different between PD and controls, only 8 genes were in common and with the same direction effect between the two sets of results. Greenbaum et al. (2003) propose at least three main reasons to explain poor correlation between mRNA and protein levels, including the multiple, complex and varied post-transcriptional mechanisms involved in turning mRNA into protein, the difference in in vivo half-lives between RNA and protein, and the significant amount of error and noise in both protein and mRNA experiments.

Nevertheless, despite this poor correlation, our proteomic analysis also revealed dysregulated proteins of interest in PD samples. Indeed, in our study, the expression of cystatin C protein was increased in PD samples and followed the same direction of expression as its gene, *CST3*. Cystatin C is an endogenous inhibitor of cysteine proteases such as cathepsins B, H, K, S, and L and is present in all mammalian body fluid and tissues (Bobek and Levine 1992). Increased expression of cystatin C in cerebrospinal fluid has been highlighted in many neurodegenerative disorders, including Alzheimer’s disease, and it was suggested to be of diagnostic interest (Deng et al. 2001; Yamamoto-Watanabe et al. 2010). In PD, Xu et al. (2005) demonstrated an overexpression of the *CST3* gene and higher levels of cystatin C in DA-depleted rat striatum. In the same line, we here describe for the first time an increased cystatin gene and protein expressions in human DA neurons of PD patients.

Recent in vitro (Kumada et al. 2004; Hasegawa et al. 2007; Tizon et al. 2010) and in vivo (Xu et al. 2005; Kaur et al. 2010) results have suggested a neuroprotective role of cystatin C. In fact, administration of human cystatin C into the rat SN*pc* partially rescued DA neurons following a 6-OHDA-induced lesion. This neuroprotective function of cystatin C may be related to its inhibitory action on cathepsins and/or to induction of autophagy.

We also observed an increased expression of cathepsin L1 in PD samples. Cathepsin L is a lysosomal cysteine endopeptidase and many in vivo studies associated cathepsins L in the maintenance of the central nervous system (Felbor et al. 2002). Cathepsins that are typically localized in lysosomes, endosomes, or vesicles could be released into the cytoplasm of degenerating neurons (Roberg and Ollinger 1998) and generate an imbalance between cystatin C (inhibitor of proteases) and cathepsins (cysteine proteases), which has been associated to Alzheimer’s disease (Nakamura et al. 1991). In 2010, Li et al. (2011) observed an abnormal cytoplasmic distribution and an increased expression of cathepsin L in DA neurons of PD patients. They also showed that inhibition of cathepsin L partially protected DA neurons from cell death induced by 6-OHDA in rodent models. Both cystatin C (Tizon et al. 2010) and cathepsins (Man and Kanneganti 2016) are involved in autophagy. Autophagy usually occurs in normal cells to preserve neuronal health by maintaining cellular turnover, clearance, and regeneration of new components. Autophagy is greatly increased in pathological contexts such as nutritional deprivation (Young et al. 2009), oxidative stress (Ciccarone et al. 2019), or hypoxia (Bellot et al. 2009). In addition, an excessive or imbalanced induction of the autophagy pathway may induce a caspase-independent form of cell death that shares many features with apoptosis (Bursch 2001; Borsello et al. 2003). According to our results, one hypothesis we may venture into is that the abnormal presence of cathepsin L into degenerating DA neurons of PD patients would induce increased expression of cystatin C, which would overactivate the autophagy pathway leading to neuronal death.

In our study, we also observed a decreased expression of Aldehyde dehydrogenase 1 in PD samples.

Encoded by *ALDH1A* gene, Aldehyde dehydrogenase 1 is a detoxification enzyme that participates in the metabolism of both dopamine (DA) and norepinephrine. It is exclusively expressed in DA neurons where it converts by oxidation a toxic metabolite of dopamine, the 3,4 dihydroxyphenylacetaldehyde (DOPAL) into a non-toxic form, the dihydrophenylacetic acid (DOPAC). In 2003, Galter et al. (2003) observed a decreased expression of *ALDH1A* mRNA in DA neurons of SN*pc* from PD patients, while DA neurons of VTA from the same patients were unaffected. Here, our study reveals for the first time a decreased expression of its gene product Aldehyde dehydrogenase 1. We could interpret this finding in two different ways. First, decreased expression of Aldehyde dehydrogenase 1 in SN*pc* DA neurons of PD patients might be a consequence of PD-related degenerative process and thus a compensatory mechanism to slow down the rate of DA-neuron degradation. Alternatively, this decreased expression of Aldehyde dehydrogenase 1 could also contribute to PD-related degeneration by allowing accumulation of DOPAL and aldehyde toxicity in DA neurons. Further studies are still necessary to appreciate whether decreased expression of Aldehyde dehydrogenase 1 may be involved in the development or the perpetuation of PD pathomechanisms.

## Conclusion

This descriptive and exploratory study is the first to generate proteomic (SI-1) and transcriptomic data (SI-2) from DA neuronal regions in PD SN*pc* and results reported above underline the potential interest of such combined molecular approaches. However, this study has also limitations, including a small set of samples and all expression changes reported above should be confirmed in more PD samples and with orthogonal approaches. Furthermore, although remaining the gold standard to decipher brain molecular alterations, autopsied tissues are associated with several drawbacks including difficulty to collect them and risks of degradation and contamination by agonal or post-mortem changes (Li et al. 2004; Crecelius et al. 2008). In fact, a massive and spreading depolarization of neurons with a high release of glutamate and potassium has been described shortly before brain death (Carlson et al. 2018). This phenomenon probably changes molecular expression in neurons, independently of PD-related events. Moreover, the post-mortem interval has also an impact on RNA and protein expression. Therefore, it may seem that the ultimate sample for research in human PD has to be safely obtained from a large number of living individuals, and sampling-to-freezer time should be kept as short as possible. Brain tissue imprints that can be collected during deep brain stimulation surgery appear promising samples for future studies using RNA sequencing or proteomics (Zaccaria et al. 2016).

## Supplementary Information

Below is the link to the electronic supplementary material.

Supplementary file1 (DOCX 284 kb)

Supplementary file2 (XLSX 521 kb)

Supplementary file3 (XLSX 1592 kb)

Supplementary file4 (XLSX 90 kb)

Supplementary file5 (XLSX 10 kb)

## Data Availability

All data relevant to the study are included in the article or as Supplementary Information. Upon reasonable request, additional information will be shared by the corresponding authors.

## Abbreviations

SN*pc*: Substantia nigra *pars compacta*
PD: Parkinson’s disease
DA: Dopaminergic neurons
LCM: Laser capture microdissection
LFQ: Label-free quantification
RIN: RNA integrity number
GFP: Gas-phase fractions
LC: Liquid chromatography
MS: Mass spectrometry
