# Supplementary material for: Multiomic Analyses of Dopaminergic Neurons Isolated from Human Substantia Nigra in Parkinson’s Disease: A Descriptive and Exploratory Study
Source: Cell Mol Neurobiol. 2021 Sep 15;42(8):2805–18. doi: 10.1007/s10571-021-01146-8 (PMC9561004; doi:10.1007/s10571-021-01146-8)
Supplement: Supplementary file 1 — Supplementary file1 (DOCX 284 kb) [file 10571_2021_1146_MOESM1_ESM.docx]

**Polyomic analyses of neuromelanin containing granules isolated from human nigral dopaminergic neurons in Parkinson’s disease.**

**Affif Zaccaria^1*^, Paola Antinori^1^, Virginie Licker^1^, Enikö Kövari^2^, Johannes A Lobrinus^3^, Pierre R Burkhard^1,4^.**

**Supplementary information**

**Supplementary Table S1.** **Samples analysed by transcriptomics with associated RIN values and number of LMD dissected Neuromelanin granules**.

Columns:

*Case ID*: C = control sample

PD = Parkinson Disease sample

*Gender*: M = male

F = female

*Age:* y = years

*PMI*: Post Mortem Delay

h = hours

*RIN*: RNA integrity Number

*Dissected NM granules*: NM = Neuro Melanin

Number of NM granules obtained by Laser Microdissection for each sample

**Supplementary Table S2.** **cDNA libraries.**

Columns:

*Case ID*: C = control sample (number 1 and 2 after the dash indicates the duplicates)

PD = Parkinson Disease sample (number 1 and 2 after the dash indicates the duplicates)

*Dissected NM granules*: NM = Neuro Melanin

Number of NM granules obtained by Laser Microdissection for each sample

*cDNA concentration (ng/µL)*: concentration of the cDNA after RNA extraction from the samples with the RNAqueous micro kit from Life Technologies, reverse transcription and cDNA amplification with the SMARTer™ Ultra Low RNA kit from Clontech. 200 pg of cDNA were used for library preparation using the Nextera XT kit from Illumina. cDNA concentration was assessed with the Qbit

*Mean size (bp):* bp = base pair

The library fragment size was assessed with the Tapestation using a DNA High sensitivity chip (Agilent Technologies). The mean fragment size and the minimum (*from*) and maximum (*to*) size are given for each library in base pairs (bp) units

**Supplementary Table S3.** **Sample analysed by proteomics and number of NM granules obtained by LMD for each sample.**

Columns:

*Case ID*: C = control sample

PD = Parkinson Disease sample

*Gender*: M = male

F = female

*Age:* y = years

*PMI*: Post Mortem Delay

h = hours

*Dissected NM granules*: NM = Neuro Melanin

Number of NM granules obtained by Laser Microdissection for each sample
